# KIAA0101 and IL2RA Were Identified as Core Genes in Hormone-Resistant Nephropathy

**DOI:** 10.1155/2022/6545266

**Published:** 2022-09-17

**Authors:** Ying Chen, Shuyi Qian, Yinyin Chen, Kanghan Liu, Wei Yin, Xun Luo

**Affiliations:** Department of Nephrology, Hunan Provincial People's Hospital, The First Affiliated Hospital of Hunan Normal University, Changsha Clinical Research Center for Kidney Disease, Hunan Clinical Research Center for Chronic Kidney Disease, Changsha 410005, China

## Abstract

**Objectives:**

To analyze the tissue heterogeneity of hormone-sensitive and drug-resistant nephrotic syndrome genes using a bioinformatics approach and to analyze gene-related functional pathways.

**Methods:**

The limma package of R software was used to screen differential genes from the nephropathy datasets GSE145969 and GSE189734. The differential genes were analyzed for functional and pathway enrichment in terms of biological processes, cellular components, and molecular functions. The Metascape tool was used to construct protein networks for the differential genes, and the results were imported into Cytoscape software for visualization. The genes were identified as key modules and genes using the MCODE plug-in. Gene set enrichment analysis was performed for the HALLMARK analysis of the two microarray key genes to obtain the relevant pathways.

**Results:**

GSE145969 screened 351 differential genes, 168 upregulated genes, and 183 downregulated genes. The differential genes were enriched in biological processes, cellular components, and molecular functions, such as myocardial contraction, intracellular nonmembrane organelles, and structural molecular activities. The protein-protein interaction (PPI) network contained 140 nodes, with the highest-scoring module containing seven genes, and the MCODE plug-in calculated the downseed. The key gene was KIAA0101, whose HALLMARK pathway was significantly enriched in the mTORC1 signaling pathway. A total of 263 differential genes were screened by GSE189734, and they were enriched in biological processes, molecular functions, and cellular components, such as immune system processes, signaling receptor binding, and the cytoplasmic matrix. The PPI network contained 253 nodes, with the highest-scoring module containing 37 genes. The seed gene obtained through the MCODE plug-in calculation was IL2RA, whose HALLMARK pathway was significantly enriched in the KRAS signaling pathway.

**Conclusion:**

By analyzing the gene sets of different tissues in nephropathy, two key genes, namely KIAA0101 and IL2RA, were obtained. Their gene function enrichment is related to cell growth, development, and reproduction. Therefore, IL2RA and KIAA0101 can be used as diagnostic markers for hormone-resistant nephropathy.

## 1. Introduction

Chronic kidney disease (CKD) is a global public health problem that will eventually evolve into renal failure and cardiovascular disease [1]. Steroid-sensitive nephrotic syndrome (SSNS) is one of the most common chronic diseases in children [2], but half of the children with SSNS will have at least four relapses per year or at least two relapses within six months after the initial visit, a condition known as frequently recurrent nephrotic syndrome (FRNS) [3]. In some of these children with FRNS, two consecutive relapses occur after a period of reduction or discontinuation of steroid therapy, a condition defined as steroid-dependent nephrotic syndrome [4]. Steroid-resistant nephrotic syndrome (SRNS) is a heterogeneous disease that includes both immune-based genes and a monogenic etiology [5]. The incidence of nephrotic syndrome is regionally dependent, with rates of 1.2–1.8 cases per 100,000 children per year in Germany [6], 3–3.5 cases per 100,000 children per year in Paris and surrounding areas [7], and 6.49 cases per 100,000 children per year in Japan [8]. Among the affected young children, males predominate, with a male-to-female ratio of 2 : 1 [9]. More than 85% of patients with nephrotic syndrome respond to corticosteroids, but about 10%–15% still do not respond to steroids or develop steroid resistance [5]. The median age of onset of SRNS is 4.4 years [10], with an early age of onset concentrated in early childhood.

With the development of bioinformatics technology, it has become an important way to obtain disease-related information for further analysis using techniques such as microarrays or multiple sequencing methods. Moreover, by grouping disease gene expression profiles for study, the causative genes can eventually be screened. The study of SSNS can be facilitated by analyzing hormone-sensitive and drug-resistant nephrotic syndrome gene-related pathways. In this study, we plan to determine the key genes of hormone-resistant nephropathy by analyzing different tissue microarrays for differential expression and gene function enrichment.

## 2. Methods and Materials

### 2.1. Sample Source

This study found two datasets related to hormone-resistant nephrotic syndrome based on the Gene Expression Omnibus (GEO, https://www.ncbi.nlm.nih.gov/geo/) gene expression databases: GSE145969 and GSE189734. The GSE145969 dataset collected data from 16 SSNS and 12 SRNS patients. The clinical information was divided into two groups: hormone sensitivity (steroid-sensitive) and hormone resistance (steroid-resistant). The GSE189734 dataset collected data on three SSNS and three SRNS patients.

### 2.2. Differential Expression Analysis to Screen for Differential Genes

The limma package of R software was used to analyze the differential expression of mRNA in the two microarrays. The results of the differential expression analysis for each microarray are shown in a volcano plot with a screening threshold of *p* < 0.05, |FC| ≥ 1.5 for differentially expressed genes (DEGs).

### 2.3. Differential Gene Function and Pathway Enrichment Analysis

In order to further study the functions of the above genes, the ClusterProfiler program package in R software was used to perform gene function enrichment analysis on DEGs to obtain gene ontological- (GO-) related pathways. GO includes Molecular Function (MF), Biological Process (BP) and Cellular Component (CC). *p* < 0.01 is statistically significant, and the results are presented as bubble plots.

### 2.4. Protein-Protein Interaction (PPI) Network Analysis

To further investigate the interaction relationship between differentially expressed genes, we performed PPI analysis on the DEGs. The DEGs were analyzed using Metascape (https://metascape.org/) to obtain the PPI relationship network. The MCODE plug-in in Cytoscape software was used to screen the important functional modules in the PPI network, select the highest-scoring cluster and seed genes in that cluster for subsequent analysis, and position the seed genes as key genes.

### 2.5. Single Gene Set Enrichment Analysis (GSEA)

Based on the median expression value of key genes, the samples were divided into two groups: high and low expressions. The HALLMARK pathway was observed using a single GSEA. The screening threshold was |*NES*| > 1, and the *p* value was set to <0.05.

## 3. Results

### 3.1. Screening of Differential Genes Using Differential Expression Analysis

**Figure 1:**
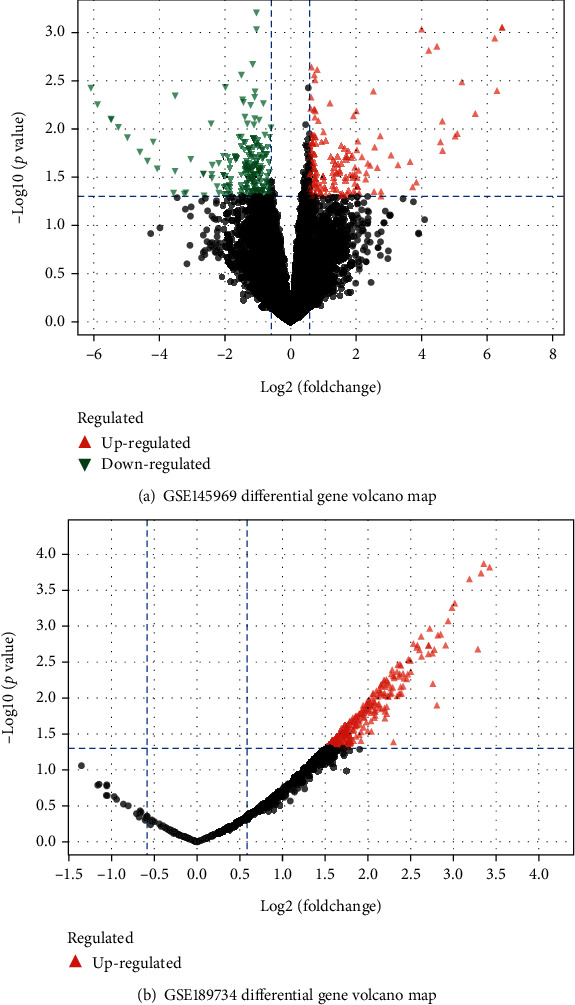
Results of the differential expression analysis. Note: The figure shows a volcano plot. The screening criteria are *p* < 0.05 and |FC| ≥ 1.5. The green portion of the figure denotes the downregulated genes, and the red portion denotes the upregulated genes.

The differential analysis of GSE145969 yielded 351 differential genes (Figure 1(a)), including 168 upregulated genes and 183 downregulated genes. The differential analysis of GSE189734 produced 263 differential genes (Figure 1(b)), which were all upregulated genes.

### 3.2. Functional Enrichment Analysis of Differential Genes

**Figure 2:**
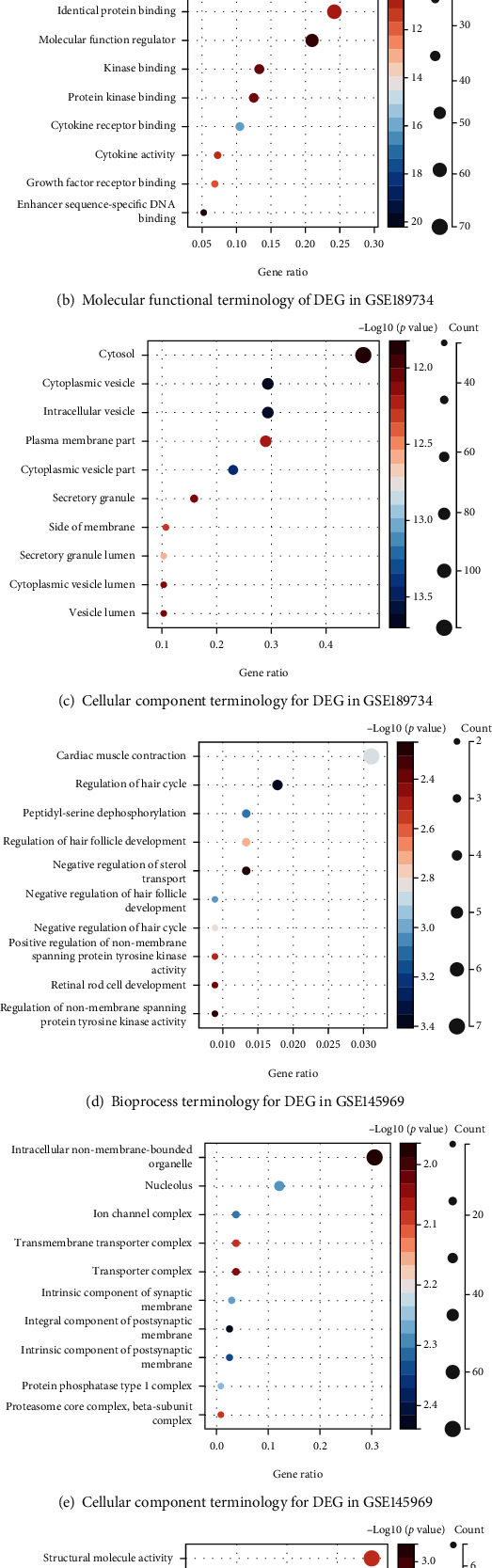
Results of the GSE189734 and GSE145969 gene functional enrichment analysis. Note: The results are shown by bubble plots for the first 10 functional pathways of genes, with a screening threshold of *p* < 0.01.

GO functional enrichment analysis was performed on the differential genes of the two microarrays. The significantly enriched biological process terms for the genes of the GSE189734 microarray included immune system processes, cellular responses to chemical stimuli, and cellular responses to organic substances (Figure 2(a)). The significantly enriched molecular function terms included signaling receptor binding and enzyme site binding (Figure 2(b)). The significantly enriched cellular component terms included cytoplasmic matrix, cytoplasmic vesicles, intracellular vesicles, and cell membranes (Figure 2(c)). The significantly enriched biological process terms included cardiac contraction and hair cycle regulation (Figure 2(d)). The significantly enriched cellular component terms included intracellular nonmembrane organelles and nucleoli (Figure 2(e)). The significantly enriched molecular function terms included structural molecular activity, ion-gated channel activity, and gated channel activity (Figure 2(f)).

### 3.3. PPI Network Analysis

**Figure 3:**
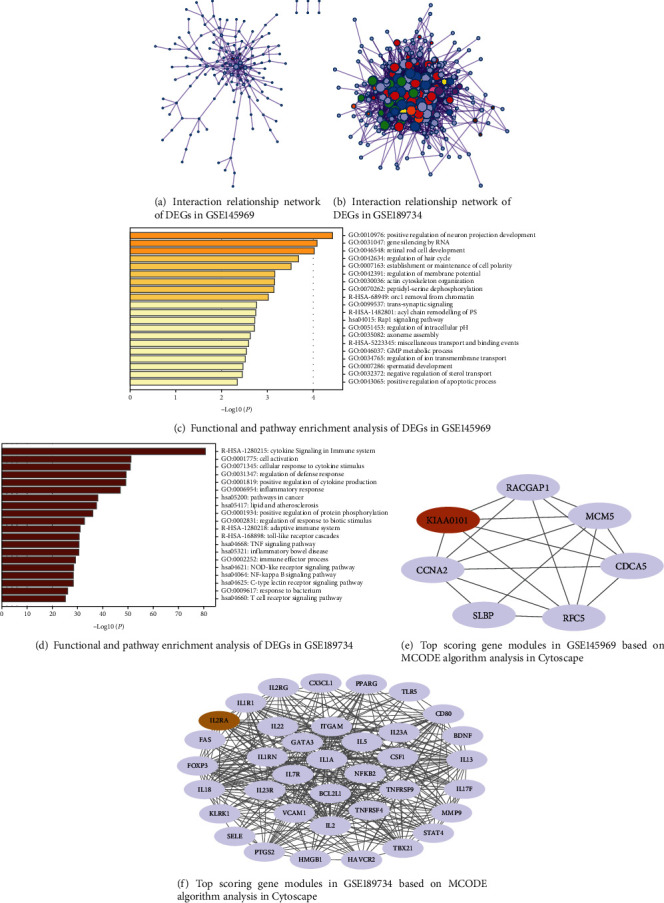
Results of the PPI analysis of GSE145969 and GSE189734. Note: As the result of the MCODE plug-in in Metascape had only one cluster and contained fewer genes, Cytoscape was used to recreate the map.

The two-dataset DEGs were analyzed using Metascape, and the PPI network constituted by the DEGs contained 140 nodes and 253 node action relationships (Figures 3(a) and 3(b)). The two datasets were analyzed for GO functional enrichment, and the results showed that the GSE145969 chip gene was significantly enriched in the positive regulation of neuron projection development and gene silencing by RNA (Figure 3(c)). The GSE189734 dataset gene was significantly enriched in cytokine signaling in the immune system and in cell activation (Figure 3(d)). The highest-scoring clusters were obtained using the MCODE plug-in in Cytoscape. The modules contained seven nodes with 18 edges and 37 nodes with 368 edges (Figures 3(e) and 3(f)).

### 3.4. GSEA of Key Genes

**Figure 4:**
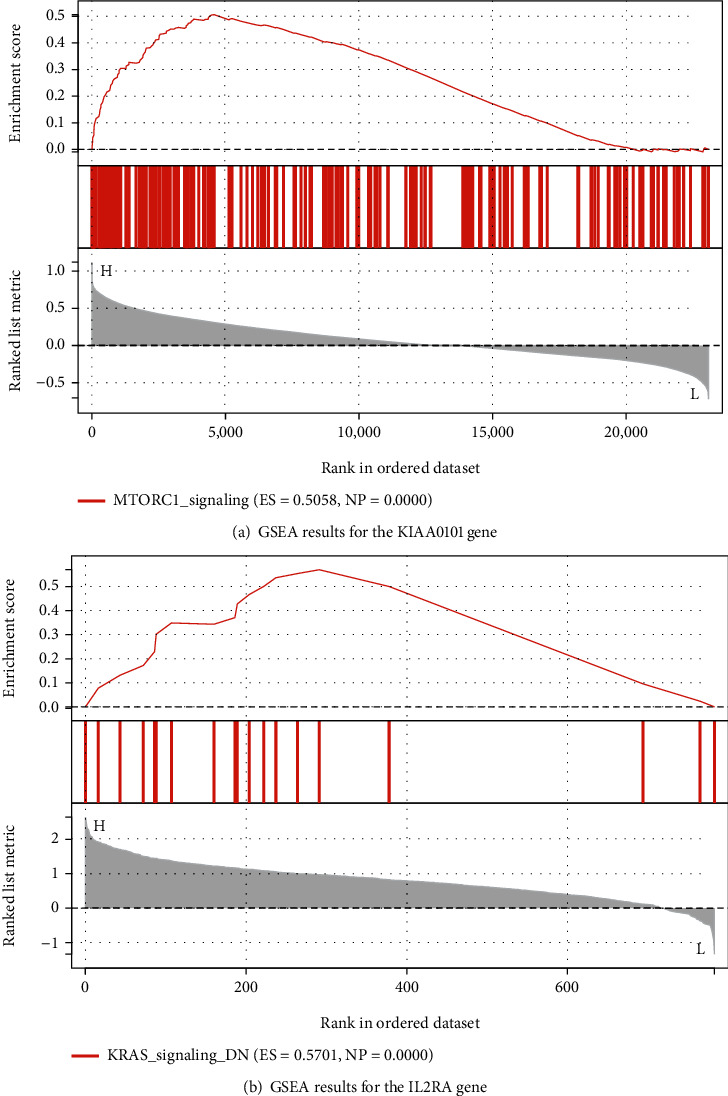
Results of the GSEA of the key genes. Note: The threshold value is |*NES*| > 1, and the *p* value is <0.05.

The key genes from the MCODE plug-in analysis were used as the key genes: KIAA0101 for the GSE145969 dataset and IL2RA for the GSE189734 chip. The GSEA of the HALLMARK pathway was performed separately. IL2RA was significantly enriched in the KRAS signaling pathway (Figure 4(b)).

## 4. Discussion

With the rapid development of biological sciences and genetics, we have gained a deeper understanding of nephrotic syndrome. In recent years, the epidemiology of nephrotic syndrome has been in a stable state and largely unchanged, but the pathology associated with it is constantly evolving. The nephrotic syndrome is reflected by urinary polyprotein, hypoproteinemia, edema, and other available clinical features, such as hyperlipidemia [11]. The pathogenesis of nephropathy is related to several factors; the most common of which is diabetic nephropathy, one of the most common microvascular complications among diabetic patients [12].

In this study, we selected different tissue samples from nephropathy for analysis, with GSE189734 containing six samples and GSE145969 containing 28 samples. Differential expression analysis was performed on the two microarrays. A total of 263 DEGs were obtained for GSE189734, and 351 genes were obtained for GSE145969, including 168 upregulated genes and 183 downregulated genes. Their functional enrichment analyses and pathways were analyzed separately. The GSE145969 dataset was functionally enriched in the positive regulation of neuron projection development. Neuron projection is established through an extremely complex transcriptional crossover, and genes operate by regulating the developmental drivers of the projection neuron subtype and another subtype [13]. Satb2 and Ctip2 are the two transcription factors that generally play a role in projection neuron development, with Satb2 acting as a repressor of Ctip2 [14]. The functions of the GSE189734 dataset are enriched in cytokine signaling in the immune system. The immune system is controlled by a variety of cytokines, which act through the Janus tyrosine kinases and the signal transduction and activators of transcription to achieve their functions [15]. In addition, the protein network models were constructed separately for the DEGs, and the genes were scored using the MCODE plug-in, resulting in the highest-scoring gene modules. The results showed that GSE189734 had 253 nodes and that the highest-scoring module contained 37 genes and 368 edges. GSE145969 had 140 nodes, and the highest-scoring module contained 7 genes and 18 edges. Clearly, the GSE189734 dataset genes were more closely related. The seed genes IL2RA and KIAA0101 were selected as the key genes for the GSEA of the HALLMARK pathway. The results showed that KIAA0101 was significantly enriched in the mTORC1 signaling pathway, while IL2RA was significantly enriched in the KRAS signaling pathway.

The KIAA0101 gene was found to be associated with the prognosis of several tumors, and its bioinformatics analysis revealed that it is an independent prognostic factor for malignant pleural mesothelioma [16]. It was also found to be a diagnostic biomarker of breast cancer prognosis in a study on breast cancer [17]. In addition, KIAA0101 is a diagnostic and prognostic marker for lung adenocarcinoma and is even associated with the gene regulatory network and immune infiltration of lung adenocarcinoma [18]. The IL2RA gene regulates proliferation, differentiation, apoptosis, and leukemogenesis and is associated with a variety of diseases, such as acute myeloid leukemia prognosis [19]. Single nucleotide polymorphisms in the IL2RA gene affect the pathogenesis of multiple sclerosis by encoding IL-2R*α* [20]. An mTOR signaling pathway is closely related to the MAPK pathway and controls cell growth by interacting to determine anabolism and catabolism. The mTORC1 signaling pathway transduces functions that regulate metabolism, translation, and autophagy [21] [22]. The overexpression of mTORC1 causes disease; therefore, mTORC1 inhibitors are used to treat various diseases [23]. KRAS is a member of the Ras family, which is a common protooncogene with a mutation rate of up to 30% [24], and is associated with a variety of cancers with poor prognoses. There are several KRAS markers in mutated cancers, such as pancreatic, colorectal, lung, and genitourinary cancers [25]. Ras proteins regulate multiple programs of cell growth, reproduction, and metabolism by signaling to pathways, such as the MAPK pathway and P13K, thus facilitating oncogenic transformation [26]. The results of the study showed that similar gene function pathways were obtained by analyzing different tissue samples from nephropathy and that all were related to cell growth, development, and reproduction.

In conclusion, hormone-resistant nephropathy was identified by two key genes, IL2RA and KIAA0101, and the signaling pathways involved were the KRAS signaling pathway and the mTORC1 signaling pathway. We hypothesize that the two genes exert their effects by influencing two signaling pathways to regulate cell growth, development, and reproduction, and that IL2RA and KIAA0101 could be used as hormone-resistant nephropathy diagnostic markers.

## Data Availability

This study found two datasets related to hormone-resistant nephrotic syndrome based on the Gene Expression Omnibus (GEO, https://www.ncbi.nlm.nih.gov/geo/)
